# A conceptual framework for sustainable waste management in small municipalities: the cases of Langebaan, South Africa and Swakopmund, Namibia

**DOI:** 10.1007/s11356-023-26904-7

**Published:** 2023-04-25

**Authors:** Timoteus Kadhila, Martin P. de Wit, Rinie Schenck

**Affiliations:** 1 Faculty of Economic and Management Sciences, School of Public Leadership, Stellenbosch University, Stellenbosch, South Africa; 2 DSI/NRF/CSIR Chair in Waste and Society, Department of Social Work, University of the Western Cape, Bellville, South Africa

**Keywords:** Circular economy, Sustainable, Challenges, Landfills, Sustainable Development Goals (SDGs), Livelihoods

## Abstract

Municipal waste management is a major challenge for local governments in South Africa and Namibia, as in other developing countries. The circular economy concept in waste management is an alternative sustainable development framework that has the potential to combat resource depletion, pollution, and poverty while achieving the SDGs. The purpose of this study was to investigate the current waste management systems in Langebaan and Swakopmund municipalities resulting from municipal policies, procedures, and practices in the context of a circular economy. A mixed method approach was used to collect qualitative and quantitative data through structured in-depth interviews, document analyses, and direct observation. The study found that the Langebaan and Swakopmund municipalities have not yet fully implemented the circular economy concept into their waste management systems. A mix of waste consisting of papers, plastics, cans, tyres, and organic products is dumped into landfills weekly at a rate of about 85%. The main challenges to implementing the circular economy concept include: lack of technical solutions, inadequate regulatory frameworks, insufficient financial resources, lack of private sector involvement, insufficient human resource capacity, and inadequate information and knowledge. A conceptual framework was therefore proposed to guide the municipalities of Langebaan and Swakopmund in implementing the circular economy concept in their waste management systems.

## Introduction

As many cities and towns around the world continue to grow due to factors such as urbanisation, industrialisation, and globalisation, waste management has consequently become a serious concern to governments worldwide (Vitharana 2015; Sharma and Jain 2020). However, governments in low-income countries spend only 20% of local budgets on waste management, and a staggering 93% of waste in these countries is either dumped or burned in the open (Wahba et al. 2019). In the twenty-first century, waste management has been a widely discussed topic around the world, due to its potential for contributing to environmental and socio-economic development. However, a lack of skills, poor infrastructure and institutional setup, and deficits in financial and technical resources have resulted in insufficient and inefficient provision for waste management at many levels (UN-Habitat 2014).

By investigating current waste management systems in Langebaan, South Africa, and Swakopmund, Namibia, this study aims to mobilise efforts seeking to improve South Africa and Namibia’s most pressing socio-economic issues, such as poverty and unemployment, in harmony with the Sustainable Development Goals (SDGs) and with circular economy (CE) principles.

A 2019 study conducted by Zamriet et al. (2019) established that waste management efforts in developing countries pose numerous challenges to the environment, the economy, and society. If waste is not properly managed, it creates environmental contamination in the form of air, water, and ground pollution, resulting in human health threats (Zamriet et al. 2019) and other significant environmental harms. For instance, as a consequence, mice, flies, mosquitos, and other animals that carry zoonotic diseases, as well as air-borne and water-borne diseases and probable cancer risks thrive in the polluted environments (Griffiths 2017; Abubakar et al. 2022). This implies that waste management is not only a technological or infrastructural subject, but also has implications for environmental, economic, and social well-being (Van de Klundert and Anschütz 2001). Fuller et al. (2022) highlight that approximately 9 million people worldwide die each year from diseases associated with different types of pollution and extreme poverty.

In addition, case studies from Brazil, Ghana, Kenya, and India have shown how supporting (and removing obstacles to) sustainable waste management systems can result in a triple win: (1) increased productivity and economic growth; (2) improved quality of employment and increased job growth; and (3) saving lives by reducing environmental impacts such as water pollution, air pollution, and climate change (Gower and Schröder 2016).

The Industrial Revolution fuelled urbanisation, which resulted in overpopulation and the escalation of towns and cities in developing countries (Boateng 2021). Eventually, a combination of mass manufacturing and urbanisation contributed to a dominant consumer culture with significant per capita waste generation (Gutberlet 2017). African countries face numerous waste management challenges, particularly with the solid-waste stream (Godfrey et al. 2019). Most African counties do not have effective waste management practices in place, due to factors such as lack of environmental awareness, lax environmental regulation and enforcement, and inadequate resources (including financial resources), all of which negatively affect human and environmental health (Godfrey et al. 2019). These challenges may hinder the implementation of a CE concept into waste management systems.

Available data in the Africa Waste Management Outlook of 2018 indicate that, in 2012, 125 million tons of municipal solid waste (MSW) were produced in Africa annually, with 81 million tons (65%) coming from sub-Saharan Africa (UNEP 2018). Furthermore, it is projected that waste generation in Africa will rise to 244 million tons per year by 2025 (UNEP 2018). For this reason, waste management in developing countries necessitates special care in waste generation (end user), waste collection, pre-processing (sorting, compressing), transportation, treatment (reuse, energy recovery, biological treatment), and waste disposal (Yu et al. 2015; Leme et al. 2014). While it is clearly important to reduce the overall amount of waste generated, there is also a need for people to capitalise on available waste products by recycling them into value-added products, thereby reducing negative environmental and socio-economic impacts in the process.

Generally, the importance of practising proper waste collection and disposal is to avoid detrimental environmental consequences and improve public well-being (Geetha and Rajalakshmi 2020). For example, anaerobic decomposition of organic waste produces greenhouse gases such as methane and carbon dioxide, which pose significant environmental hazards including climate change and global warming (Cunha-Santino et al. 2016). By contrast, as previously highlighted, transitioning to sustainable waste management could have substantial positive environmental and socio-economic consequences, such as the creation of jobs in recycling industries (Wahba et al. 2019). Unfortunately, waste collection services in developing towns and cities are frequently adequate only in city centres, while waste collection services in outskirts and peri-urban areas are typically inadequate (Rai et al. 2019). The costs of waste management activities coupled with the lack of large-scale infrastructure and technology interventions are also heavy burdens for small municipalities in developing countries (Mihai and Taherzadeh 2017).

To overcome socio-economic issues such as unemployment and pollution, it is necessary to establish a sustainable economy by re-examining current patterns of production and consumption of products (Wahba et al. 2019). This can be achieved through dematerialisation, remanufacturing, and recycling, which are some of the CE practices that promote sustainable waste management (UNEP 2008). Generally, various waste streams are reusable and compostable, which can generate income if managed appropriately in the global market (Srivastava and Singhvi 2013). Hence, many people recognise waste as a potential secondary resource that should be handled with care to ensure both short- and long-term resource availability (Parizeau 2016). Informal waste sectors, which are most prevalent in African countries, view waste as a substantial source of income that has already improved many livelihoods (Godfrey et al. 2019). Again, making new products is often an expensive and energy-intensive process, which is why participants in the informal waste sector opt for waste recycling and reusing (Srivastava and Singhvi 2013). Recycling and reusing of waste materials are activities that are already making a significant and widespread contribution to sustainable production and resource efficiency.

Saita and Franceschelli (2017) emphasise that other waste management strategies should be employed as an alternative to traditional landfill disposal, with waste materials being retrieved and recycled as a secondary resource to achieve SDGs and a CE. Applying the CE model to waste management has many advantages, as it encompasses waste reduction, employment creation, and income generation (Cook, Smith and Utting 2012). In addition, a CE places a renewed emphasis on the economy, investment, capital, infrastructure, and skills development, all with positive social and economic outcomes (United Nations Environment Programme [UNEP] 2021).

One of the key objectives of this study is to investigate how to improve basic human conditions in line with SDG1 (no poverty), SDG3 (good health and well-being), SDG8 (decent work and economic growth), and SDG11 (sustainable cities and communities), by fostering CE infrastructures and capacity-building. In addition, the study is mindful about the natural environment and natural resources in line with SDG12 (responsible consumption and production) through decoupling development from the use of virgin natural resources. Therefore, the conceptual framework developed by this study is intended to influence policy decisions in small municipalities to ensure waste management practices that benefit society and promote sustainability.

**Fig. 1:**
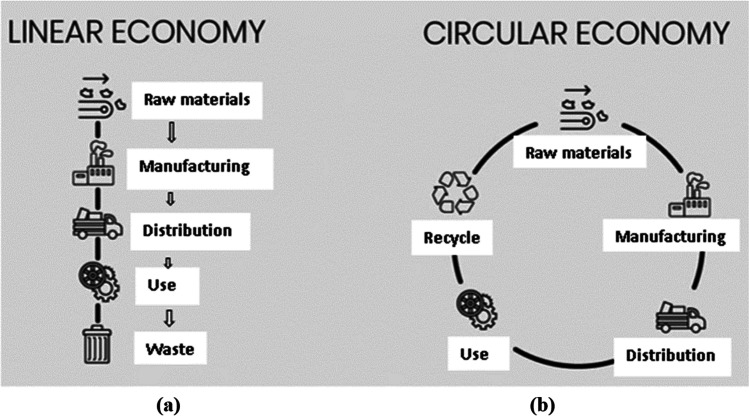
(**a**) The linear economy and (**b**) circular economy models. Source: Ezeudu and Ezeudu (2019)

Based on the literature studied, the CE model developed by Ezeudu and Ezeudu (2019) and Ellen MacArthur Foundation (2013) was selected as the most appropriate conceptual framework to underpin this study. The CE model provides an in-depth understanding of the extent to which the current waste management systems based on the municipal policies, processes, procedures, and practices in the municipalities of Langebaan and Swakopmund are contributing to the realisation of sustainable waste management. Figure 1 illustrates the different stages of the CE model as opposed to the dominant linear economy (LE) model.

Figure 1 above presents two scenarios, namely the CE and the LE models. The LE model is represented in Fig. 1 (a), while CE model is represented in Fig. 1 (b). To date, most municipalities design their waste management systems based on the LE model (Gower and Schröder 2016).

In the LE model as presented in Fig. 1 (a), waste handling modalities view waste products as nuisance that often entails the disposal of scarce resources as a management approach. In this model, the premise is that companies extract raw materials, apply energy to them to manufacture a product, and sell the product to an end consumer, who then discards it when it no longer works or no longer serves the user’s purpose (Ellen MacArthur Foundation 2013). This inevitably implies that the LE model causes unnecessary resource losses in the production chain and causes damage to natural ecosystem. For example, during manufacturing, companies produce large volumes of materials such as parting materials that are not physically incorporated into the formal economic system but disposed of at landfills and dumpsites. Gower and Schröder (2016) pointed out that in Europe, for example, an average of 95% of a product’s materials and energy value is wasted in this way.

In contrast, the CE is a system of production and consumption based on reusable and sustainable design (Ellen MacArthur Foundation 2013). It seeks to eliminate waste from the current and dominant linear production system, also known as “take-make-consume-waste”, whereby raw materials are extracted and disposed of not long after (Ezeudu and Ezeudu 2019).

Globally, LE models of waste management have increasingly been criticised by economic and environmental pundits for being counterproductive in promoting job opportunities and for causing resource depletion, while CE models are cited as presenting more sustainable solutions for waste management. Therefore, the purpose of this study was to explore the current waste management systems based on municipal policies, processes, procedures, and practices of the Langebaan and Swakopmund municipalities, and to use the results to contribute to a framework for more sustainable waste management (environmental, economic, and social sustainability), guided by the concept of the CE. This includes comparing the landfilling method of waste management to methods such as reducing, reusing, and recycling, with the aim of reducing waste and improving livelihoods. Due to high unemployment rates in both South Africa and Namibia, implementation of the CE model by the Langebaan and Swakopmund municipalities would enable them to enhance resource efficiency, create job opportunities, and improve the livelihoods of the communities through successful waste management practices.

In the Netherlands, for instance, the concept of the CE is achieved through the following core strategies: (1) prioritising regenerative resources (ensuring renewable, reusable, non-toxic resources are utilised as materials and energy); (2) using waste as a resource (utilising waste streams as a source of secondary resources and recovering waste for reusing and recycling); (3) preserving and extending what is already made by maintaining, repairing, and upgrading; (4) incorporating digital technology to track and optimise resource use and strengthen connections between supply chain actors through digital and online platforms (Douma et al. 2015). This study thus developed a framework for waste management applicable to municipalities in South Africa and Namibia, and benchmarked by some of these international good practices.

## Methodology

This study employed a comparative exploratory case study design to explore and compare the extent to which the current waste management systems based on the municipal policies, processes, procedures, and practices in the municipalities of Langebaan and Swakopmund are contributing to the realisation of sustainable waste management (environmental, economic, and social sustainability) within the framework of a CE.

A mixed method approach that incorporated qualitative and quantitative data collection approaches was used in this study. Structured in-depth interviews, document analysis (such as of policy documents and environmental management plans), and direct observation were methods employed to collect data. Participants were senior-level employees at Langebaan and Swakopmund municipalities as well as private waste management companies.

The participants were purposely selected on the basis of being knowledgeable about the waste management systems in their respective municipalities. Video-conference links on the virtual platform Zoom were created and shared with participants from both municipalities. Individual in-depth interviews were conducted using the Zoom virtual platform as, at the time of data collection, the study needed to observe social distancing protocols due to the COVID-19 pandemic.

Structured in-depth interviews contained predetermined questions covering the collection of both qualitative and quantitative data. The interviews lasted 25–30 min. The interview questions were mostly open, leaving space for the researcher to probe further by asking follow-up questions. It facilitated two-way communication by allowing participants to respond with open-ended questions or statements for more in-depth information and understanding (Creswell 2013). Research questions were built around current municipal waste management policies, processes, procedures, and practices, and sustainable waste management and CE application in the two municipalities.

Document analysis was used to conduct a reflective/comparative scrutiny of documents (such as policies, procedures, processes, organisational strategies, and reports) as ways of providing additional data and for triangulation and confirmation of validity of the data collected through interviews. Documents provided background information and broad coverage of both qualitative and quantitative data and were therefore helpful in contextualising the research (Brannen 2005). Participants were requested to provide policy documents, environmental management plans (EMPs), and other available documents for analysis.

Direct observation (landfill site visits) was conducted to observe, evaluate, and establish the current practices and operations of waste management such as waste disposal methods and recycling activities. A thematic analysis approach using the ATLAS.ti 8 package was employed to identify, analyse, and report themes (patterns) within qualitative data sets, while quantitative data analysis generated descriptive statistics including frequencies and percentages.

Ethical issues were addressed in the course of the data collection process, including informed consent, confidentiality, and anonymity. Ethical approval for the research was given by the University of Stellenbosch. The purpose of the study was explained to participants and they were made aware that participation was optional. In both municipalities, participants were labelled as P1-7.

### Study area

**Fig. 2:**
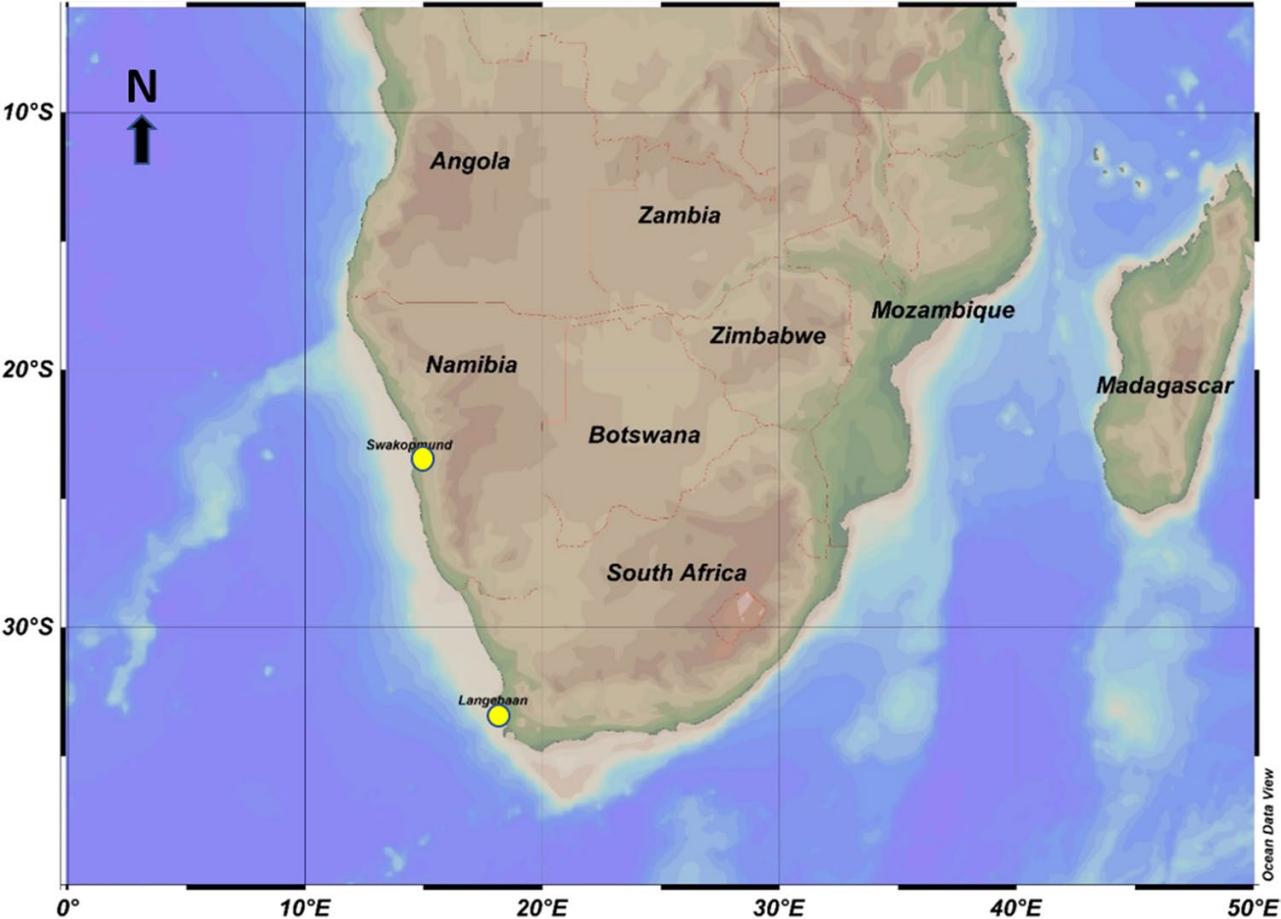
Langebaan and Swakopmund

Figure 2 shows the location of Langebaan and Swakopmund towns in Southern Africa.

Langebaan is a coastal town in South Africa, situated in the Saldanha Bay Municipality of the Western Cape Province, about 120 km north of Cape Town. According to the 2011 census, it has a population of 8297 people (City Population 2018; Alli n.d.). In 2019, Saldanha Bay Municipality’s total workforce was estimated to consist of 51,433 workers, with 39,343 (76.5%) working in the formal sector and 12,090 (23.5%) working in the informal sector (Western Cape Government [WCG] 2020). In addition, the municipality’s unemployment rate was recorded as 22.4% in 2011 and 17.4% in 2019, the highest in the entire West Coast District (Western Cape Government [WCG] 2020). Despite being significantly higher than the district average (11.9%), it was significantly lower than the Western Cape’s overall unemployment rate of 19.4% (Western Cape Government [WCG] 2020).

Swakopmund is Namibia’s central coastal town and a renowned holiday destination for Namibians and foreigners (Swakopmund Municipality 2019). During the Namibia’s 2011 national census, the Swakopmund population was 44,725 with a labour force of 25,812 workers and an unemployment rate of 25.6%, higher than the 22.6% Erongo regional average (Swakopmund Municipality 2019).

This study intends to explore initiatives that have the potential to reduce unemployment in Langebaan and Swakopmund when the CE concept in the waste management system is applied. In addition, the study aims to address a lack of empirical data on CE job opportunities in the context of small municipalities such as Langebaan and Swakopmund.

## Results and discussion

### Existing waste management systems in Langebaan and Swakopmund municipalities

**Fig. 3:**
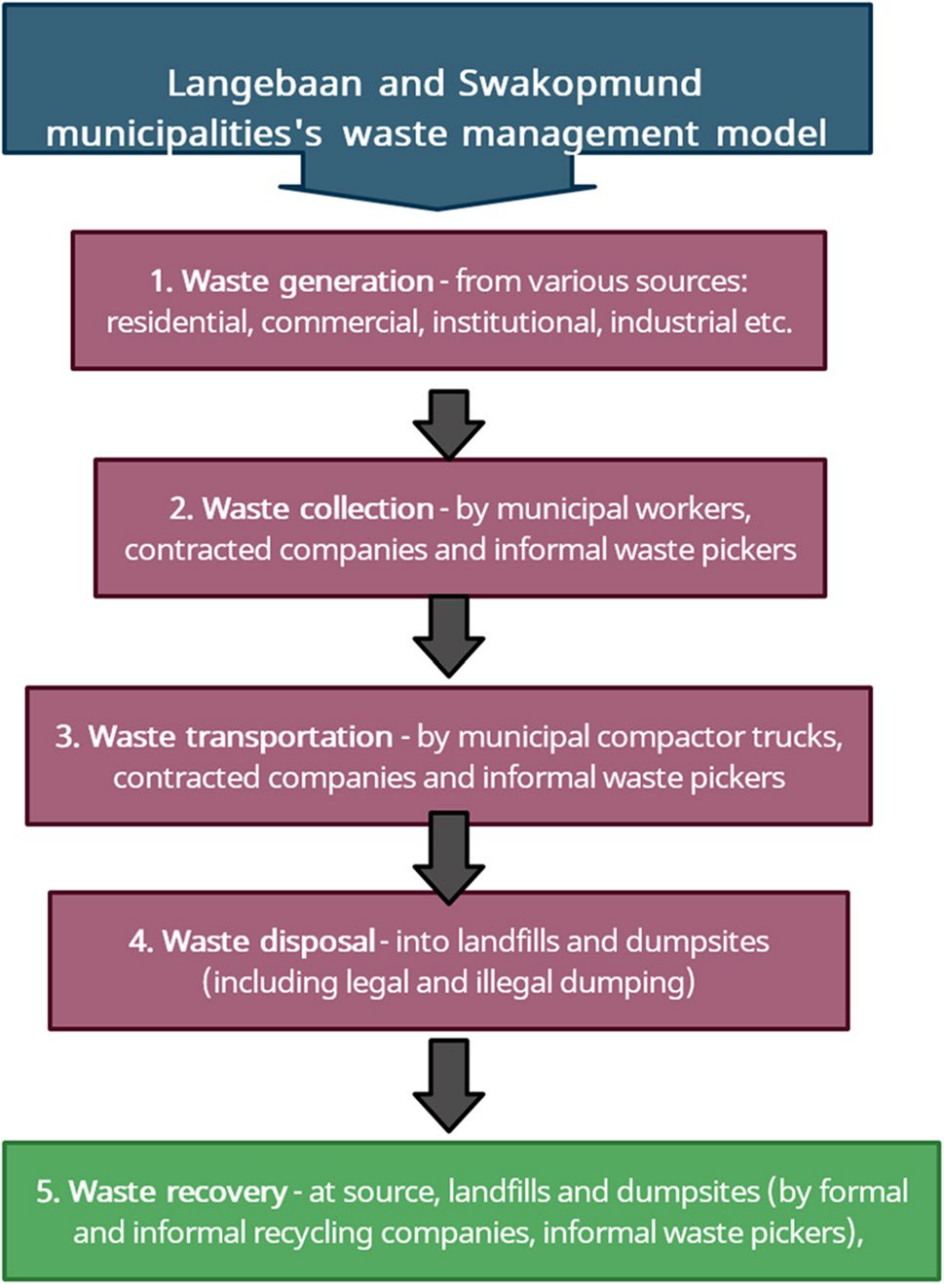
Waste management system in Langebaan and Swakopmund

First of all, the study established what the existing waste management systems in the two municipalities are like. Figure 3 depicts the researchers’ understanding of the similarities between the current waste management systems used by Langebaan and Swakopmund, based on the results of structured in-depth interviews with participants from the two municipalities. A municipality’s waste management system emerges from its municipal waste management policies, processes, procedures, and practices.

Figure 3 depicts how the two municipalities use a similar waste management system in which municipal waste is collected by municipal employees or contractors. The waste is then transported from residential and commercial areas by waste trucks and disposed of as mixed waste at approved municipal landfill sites. However, there are also some waste management companies who collect and transport mixed waste to their material recovery facility (MRF), where recyclable waste materials are recovered. In general, the municipalities of Langebaan and Swakopmund have not yet fully implemented the CE model in their waste management systems.

### Existing waste management methods in Langebaan and Swakopmund municipalities

Fourteen (14) employees (seven (7) from each municipality) were interviewed to provide information about the waste management methods used in the two municipalities. These participants are familiar with and knowledgeable about their respective municipalities’ waste management systems and were therefore interviewed to collect “rich” information. Their responses are summarised in Table 1.

**Table 1:** Types of waste management methods in Langebaan and Swakopmund

Methods	Langebaan	Swakopmund
	Participants (Yes)	Participants (Yes)
Reduction at source – *any action that reduces or eliminates the generation of waste at the source, usually within a process*	P7, P2, P5	P3, P4
Reusing – *using products or components that are not waste again for the same purpose*	P1, P2, P3, P4	P3, P4, P5
Recycling – *converting waste products into new products, or processing discarded items to extract or recover materials and resources*	P1, P2, P3, P4, P5, P6	P3, P4, P4, P7
Recovering – *taking useful discarded items for a specific next use*	P1, P2, P3, P4, P5, P6	P1, P2, P3, P4, P5
Remanufacturing – *rebuilding or recovering previously sold or worn, or non-functional products*	P7	P2
Composting – *natural bio-degradation process that takes organic wastes such as the remains of plants and garden and kitchen waste and turns them into nutrient-rich food for plants*	P7	0
Repairing – *restoring to a good or sound condition after decay or damage*	P3, P4	P7, P3, P5
Landfilling – *burying waste in the land or throwing daily waste/garbage in landfills*	P1, P2, P3, P4, P5, P6, P7	P1, P2, P3, P4, P5, P6, P7

As seen in Table 1, all of the participants from both municipalities indicated that the landfilling method is used in both municipalities, indicating its prevalence. In Langebaan, six (6) participants indicated that waste recycling and recovering occurs in the municipality, four (4) participants indicated the presence of reusing, and three (3) indicated that waste reduction at source occurs. In comparison, in Swakopmund, four (4) participants indicated the use of recycling, five (5) indicated recovering, three (3) participants indicated reusing, and two (2) participants indicated waste reduction at source. Only one (1) participant in both municipalities indicated that remanufacturing of waste materials occurs (remanufacturing of plastics, cardboard boxes, and old furniture). One (1) participant in Langebaan indicated that waste composting occurs in that municipality, while in Swakopmund none of participants indicated that waste composting occurs. The study findings indicate that there is a level of waste diversion from landfills occurring in the two municipalities through sustainable waste management practices such as waste recovering, reusing, and recycling activities. However, these practices should be promoted in order to fully minimise waste generation and reduce the level of waste landfilling.

### Langebaan’s Integrated Waste Management Policy and Integrated Waste Management Plan

Participants were requested to provide policy documents and other associated documents such as waste management plans to assess the municipalities’ readiness to implement sustainable waste management. Based on the documents acquired, Langebaan’s waste management system is governed by an Integrated Waste Management Policy (IWMP) (Saldanha Bay Municipality 2016) and the Integrated Waste Management Plan (Saldanha Bay Municipality 2017). Generally, identifying the objectives of the policy is of great importance and interest in terms of governance and management of the municipality, and in determining its success in waste management. Table 2 provides an analysis of Langebaan’s IWMP of 2016 and Integrated Waste Management Plan of 2017, based on the interviews and document analysis.

**Table 2:** Saldanha Bay Municipality—Langebaan’s Integrated Waste Management Policy and the Integrated Waste Management Plan

Specific objectives	Key achievements (interviews and document analysis)	Main challenges (interviews and document analysis)
1. Ensure waste is managed in an integrated manner.	• Development of integrated waste management plan. • Compiling of by-law for waste management (for example, levy of the use of plastic shopping bags).	• Lack of compliance and enforcement. • Ineffective policy monitoring. • Limited job opportunities. • Limited stakeholder participation. • Limited knowledge. • Lack of adequate data. • Limited resources. • Limited expertise.
2. Promote waste avoidance and minimisation.	• Inception of separation at source programme.	• Limited control and monitoring measures. • No strict enforcement to separate recyclables. • Limited public/private partnership. • Limited public awareness.
3. Facilitate and encourage separation of waste into different streams at source by all waste generators, including households, government, and business.	• Incentives to engage in recycling provided to households.	• Does not prohibit the storage of recyclables (for example, plastics, metals, glass, paper, cardboards) in landfills.
4. Facilitate diversion of recyclable and reusable waste from landfill to ensure that all waste generators receive a service from a legitimate waste service provider according to the municipality’s statutory obligations and prerogatives.	• Material recovery facility established. • Fraction of recyclable materials recovered. • Provision of waste management services by certified contractors.	• Low waste diversion rate from landfill because landfill still cheap option for waste disposal. • No model approach is identified that could accelerate transformation to CE. • Lack of facilities and technology. • Lack of incentives (for example, financial) mentioned in the policy to support informal circular entrepreneurs. • Low number of formal processing and recycling businesses.
5. Ensure that waste management processes and methods practiced within the municipality are not harmful to the environment or human health.	• Minimum negative environmental impacts. • One of the cleanest and competitive municipalities in the country. • Measures to protect environment and human health introduced by the policy.	• Amount of waste generated is not decreasing. • Waste management did not limit the overall effects of resource use.
6. Promote local economic development by capitalising on the economic value in waste.	• Waste recovery facilities established.	• Recovered waste material exported lack of initiatives. • Waste processing infrastructures privatised. • Shortage of processing infrastructure and skilled staff.

The results (Table 2) show that Langebaan municipality ensure that waste is managed in an integrated manner, [strive to] promote waste avoidance and minimisation, facilitates and encourages separation of waste, [strive to] facilitate diversion of recyclable and reusable waste from landfill, ensures that methods practised within the municipality are not harmful to the environment or human health, and promotes local economic development.

It can be observed (Table 2) that the policy and plan are intended to promote formal job creation and livelihood improvement in the waste management setup. For instance, waste separation at the source programme was incepted in the Saldanha Bay–Langebaan Municipality to promote formal job creation. The material recovery facilities that were established provide an enabling environment for waste recovery and recycling. However, the policy and plan did not mention promoting informal waste pickers. The policy and plan make provisions for certified contractors to provide waste management services to the communities, therefore promoting the formal waste sector. This practice provides job opportunities to the private sector and promotes public/private partnerships (PPP) in the waste sector. The policy did not specify that certification of contractors also includes small and mid-size enterprises (SMEs), or only large companies. In fact, the policy seems to constrain job opportunities among the informal waste workers; one of the participants from Langebaan P1 stated that “*the policy does not allow informal waste pickers to operate from the landfill site”*.

### Swakopmund’s Waste Management Policy

The Swakopmund municipality’s waste management system is governed by the Waste Management Policy of 2015 (Council of Swakopmund [CoS] 2015). Table 3 summarises the key achievements and the main challenges within the context of the Swakopmund Municipality’s Waste Management Policy, based on the interviews and document analysis.

**Table 3:** Swakopmund municipality’s Waste Management Policy

Specific objectives	Key achievements (interviews and document analysis)	Main challenges (interviews and document analysis)
1. To improve the standard of living for all residents and visitors by encouraging the avoidance, reduction, reuse, and recycling of waste to eliminate any environmental impacts associated with waste generation and waste handling.	• In line with SDGs 3 and 12 and Global Agenda 21. • Waste is being avoided and reduced (for example, the introduction of levy for reducing the use of plastic bags). • Reusing of items is encouraged. • Recycling is currently taking place in the private sector. • Environmental impacts are minimal. • Waste management strategies are based on CE principles.	• Limited incentives to SMEs. • Low rate of CE activities (waste avoidance, reduction, reuse and recycling). • No specific limitation for waste generated – large volumes of waste are still going to landfill. • The municipality does not own a recycling, remanufacturing or composting facility, hence private sector more active in recycling. • Informal waste workers do not get sufficient support such as waste-sorting equipment. • Limited law enforcement – illegal dumping still persists.
2. To develop mechanisms to encourage the private sector to enter into solid waste management operations and to formally connect the public and private sectors towards improving the effectiveness and efficiency of management of waste.	• Registered private companies are tendered to provide waste collection services.	• Mechanisms to encourage the private sector into solid waste management operations are not specified. • Lack of funds for SMEs. • Lack of sufficient waste processing infrastructure. • Circular economy concept is not mentioned in the policy. • The role of local economic development (LED) is not specified. • Lack of incentives. • Lack of public awareness.
3. To identify responsibilities of council, stakeholders and residents to ensure: a) a clean environment; b) the collection and/or transport of waste to appropriate waste facilities; c) processing or treatment of waste for recycling and reuse, if possible.	• Clean environment. • Collection and/or transport of waste to appropriate waste facilities.	• Processing or treatment of waste for recycling and reuse is minimal. • The ambitious target for increasing the reuse and recycling of waste materials is not indicated (for example, the minimum target).
4. To promote a sense of responsibility through environmental education and environmental awareness programmes.	• Environmental education and environmental awareness programmes.	• Less environmental education and fewer environmental awareness programmes. • Lack of published CE action plans.
5. To provide an effective service at an economically acceptable rate.	• Effective service takes place in the formal settlement at acceptable rate.	• No effective service in the informal settlement.
6. To ensure fair cost norms (framed by the availability of funds and as per the municipal tariffs).	• Formal property owners pay the costs of providing waste management services such as waste collection.	
7. To establish the IWMP of the council, based on waste minimisation and sound socio-economic and environmental principles.	• IWMP drafted.	• Since 2015, the approval of an IWMP is still pending. ••••••Lack of IWMP makes waste monitoring difficult (for example, recycling rate, and waste generation per capita). • Lack of CE implementation plans (roadmaps) at municipal level.

The results (Table 3) show that the Swakopmund municipality improve the standard of living for its residents and visitors by encouraging the avoidance, reduction, reuse, and recycling of waste; develop mechanisms to encourage the private sector to enter into waste management operations; identifies responsibilities of council, stakeholders, and residents to ensure a clean environment and promotes a sense of responsibility through environmental education and environmental awareness programmes. Table 3 further indicates that the objectives of the policy strive to provide high-quality services at reasonable costs, to ensure fair cost norms and lastly to establish an IWMP of the council, based on waste minimisation and sound socio-economic and environmental principles.

The analysis of the waste policy in Table 3 shows that the policy is promoting job creation in many ways. It is indicated that waste recycling businesses are increasing, particularly in the private sector, a position that provides promising job opportunities to society. The policy also makes provision for private waste management contractors to provide waste management services in the municipality. It was also confirmed that the policy gives the opportunity to informal waste workers to recover sufficient waste and gain a reasonable income; however, only a limited number of informal waste workers are permitted to recover waste materials inside the facility.

### The benefits of implementing circular economy model in waste management

Implementing CE models for waste management in Langebaan and Swakopmund municipalities, or any other business environment, will offer environmental, economic, and social benefits. During the interviews, participants from Langebaan and Swakopmund identified several environmental, economic, and social benefits that CE may offer.

The economic benefits include (cited by participants: Langebaan P1, P2, P4, P6, P7; Swakopmund P1, P2, P3, P4, P5, P6):

*“Reducing the use of primary resources (production) through recycling, resource efficiency, and the utilisation of renewable energy sources”*; *“There are opportunities for job creation in shifting waste away from landfilling and toward alternate waste treatment”*; *“Keeping materials and goods at their best value (production) through remanufacturing, refurbishment, and reuse of items and components, as well as product life extension”*; *“Changing consumption patterns through sharing models, resulting in a shift in consumption patterns”*; *“Use of recycled materials in manufacturing processes”*.

Environmental benefits include (cited by participants: Langebaan P1, P2, P3, P7; Swakopmund P1, P2, P3, P4, P5):

*“Reduced environmental pollution from production and consumption activities”*;* “Reduced use of landfills and incineration”*; *“Fewer emissions and raw materials extraction”.*

Social benefits include (cited by participants: Langebaan P4, P5, P6, P7; Swakopmund P1, P2, P3, P4):

*“Livelihood improvement”*;*” Food waste: redistribution of edible food”*; *“Waste reduction and recycling in health sector”; “Job opportunities”*

The benefits identified by participants corresponds to those in the published literature. For example, Jørgensen and Pedersen (2018) point out that investing in CE could potentially create jobs and reduce unemployment rates. The abovementioned CE benefits should be priority areas of CE strategic plans where CE projects are possible. The CE will be of assistance to maximise value and reduce waste through improving and reforming the way goods and services are designed, made, and consumed. It covers everything from material selection and company strategy to regulatory frameworks, incentives, and market design. For example, by introducing recycling points across municipalities as part of recycling schemes and projects should divert more waste from landfills.

### Current employment in the waste management sector

In the structured in-depth interviews, participants in both municipalities were asked to indicate the number of people employed in their waste management sector, in order to gain a better understanding of job creation in this sector.

**Fig. 4:**
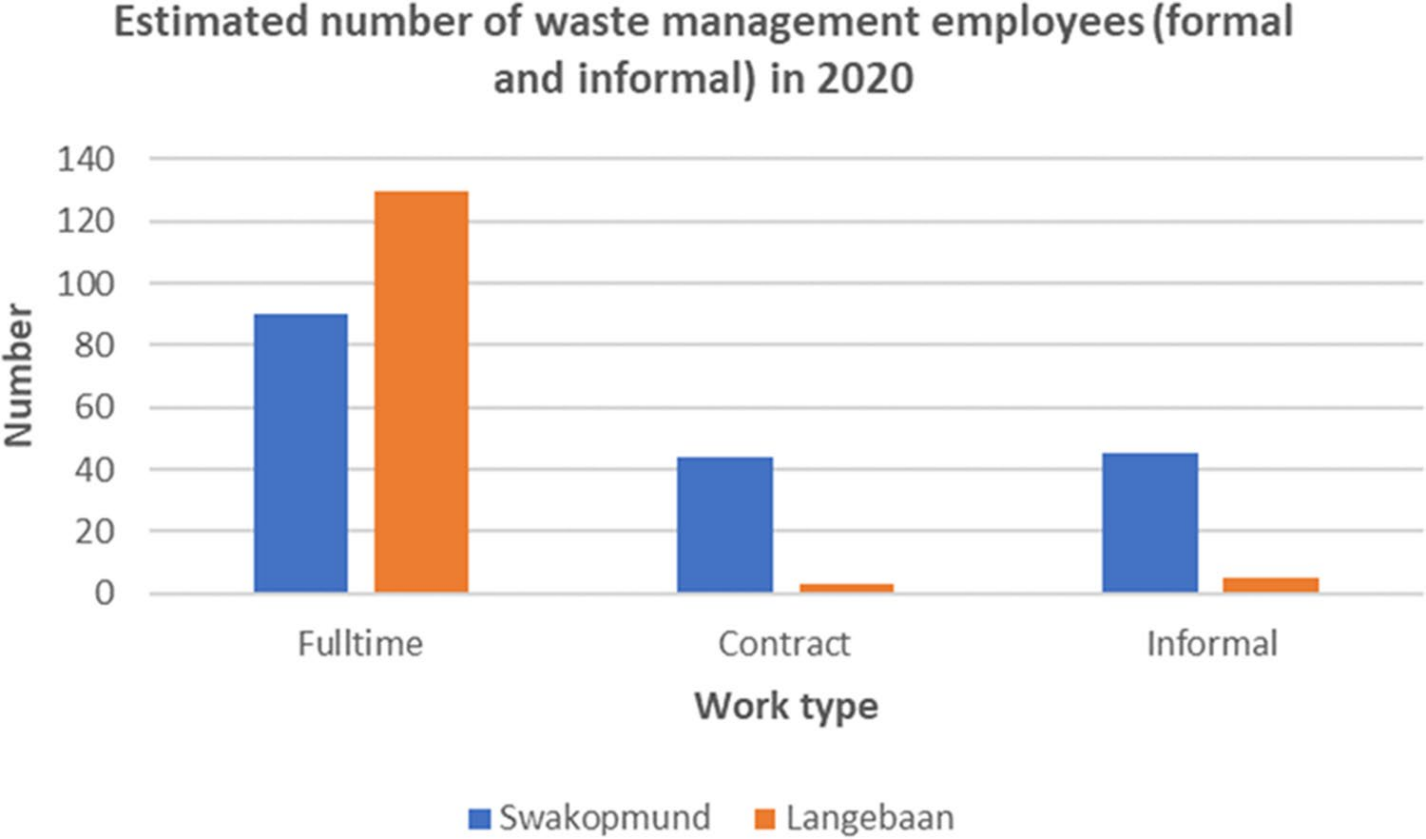
Number of employees working in waste management per municipality

The results (Fig. 4) show that the Langebaan municipality has approximately 130 full-time employees, whereas the Swakopmund municipality has about 90 full-time employees and 44 ward cleaners on a contract basis. The number of contract waste workers in Langebaan municipality is estimated to be fewer than 5, while in Swakopmund, the number is estimated to be just over 40. In Langebaan, fewer than 10 informal waste workers are estimated to recover waste products on a daily basis, which in Swakopmund there are estimated to be more than 40 informal waste workers. The quantity and quality of job opportunities may increase if CE is implemented in the entire production and consumption chain whereby waste products are taken back into the economy at the waste source, for example if recycling takes place in the production plants and no waste goes to landfill.

### Negative impacts of waste dumping

**Fig. 5:**
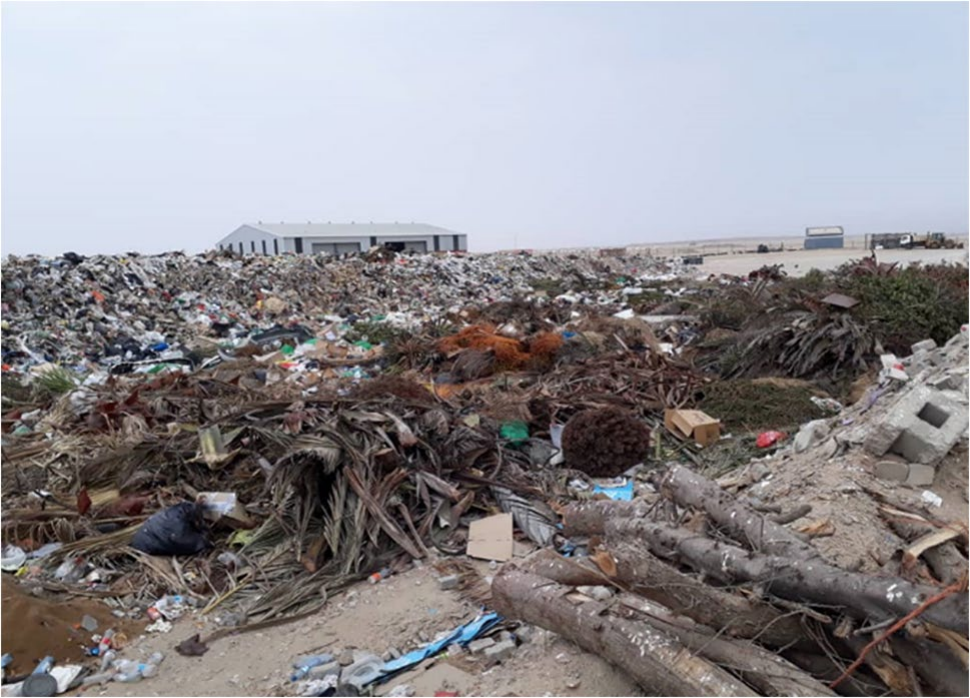
Waste disposal in Swakopmund

Figure 5 shows the impacts on human health related to poor waste management, which causes air pollution, land pollution, and water pollution. All of these have both direct and indirect negative impacts on human health.

Pollution is highly associated with unregulated waste disposal and has harmful consequences to natural environments (Hayes 2016). Direct observation in Swakopmund municipality shows that there is a high degree of waste dumped into the landfill (Fig. 5). The size of the landfill in Fig. 5 is about 4 ha and receives approximately 10 tons of waste materials daily. Based on direct observation, public health issues include flies, which carry pathogens on their bodies and spit them out into the environment. Malaria-transmitting mosquitoes also breed in waste materials such as cans and tyres. Based on the latest World Malaria Report, there were 247 million cases of malaria in 2021 compared to 245 million cases in 2020 (World Health Organisation 2022). The estimated number of malaria deaths was 619,000 in 2021 compared to 625,000 in 2020 (World Health Organisation 2022). In 2020, South Africa recorded 64,622 malaria cases and 38 deaths, while Namibia recorded 20,000 cases and over 50 deaths nation-wide (World Health Organisation 2022). It is therefore a serious health concern associated with waste management.

No waste segregation is taking place during waste dumping and a mix of waste materials is usually dumped in the landfill, as seen in Fig. 5. Hence, there is a low level of CE activities such as recycling and reusing. Illegal dumping and burning of waste materials also occur mainly in informal settlements, where there is inadequate formal waste collection services, posing health hazards to residents.

### Skills needed to implement the circular economy

Even though both municipalities have a number of employees, it was important to determine whether the skills needed to implement CE principles in the waste management systems are present among employees. The results and findings are presented in Table 4, which shows responses from participants indicating that law enforcement, education, and awareness are viewed as the skills most needed to implement the CE.

**Table 4:** Skills to implement CE

No	Skills	ID
1	Law enforcement	Langebaan P1, P3, P7; Swakopmund P1, P4, P6, P7
2	Education and awareness	Langebaan P2, P4; Swakopmund P5, P6
3	Modern high-tech machinery operation	Langebaan P7; Swakopmund P2, P5
4	Marketing of emerging circular businesses	Langebaan P3, P7; Swakopmund P3
5	Communication	Langebaan P4, P5; Swakopmund P6

Based on the responses from participants (Table 4), law enforcement skills enable them to solve and manage waste problems. Participants also pointed out the importance of education and awareness skills that enable general learning about best waste management practices. In addition, participants indicated the significance of modern high-tech machinery operation skills that provide knowledge required in material production. Marketing of emerging circular businesses is also a necessary skill that enables stakeholders to sell circular products while communicating with other stakeholders in the market.

### Drivers and barriers of sustainable waste management

Document analysis, coupled with interviews, revealed that transitioning to a CE model requires the following key drivers or enablers: *“The society’s mind-set should change; provide waste management education; innovate and use technology; establish corresponding waste management infrastructure”* (Langebaan P5). A number of key drivers that contribute towards a CE model were mentioned by several participants. Langebaan P2 stated *“preparing, implementing and developing new business and market models; developing waste management systems; change in priorities and habits of consumers, as well as development of new behaviour patterns and developing new methods for management of integrated systems.”* The responses from Swakopmund P5, P2, and P3 stated that some of the key drivers required are: *“developing new financial products supporting the concept of CE; and defining and communicating new policies.”* Table 5 summarises the responses from participants.

**Table 5:** Enablers/drivers of and barriers to sustainable waste management in Langebaan and Swakopmund

Enablers/drivers	Barriers
Societal organisation (user’s active role)	Costs: running capital
Education and awareness (knowledge sharing)	Legislations: lack of CE policies and implementation
Innovations	Logistics: weak logistics in the material flow
Technology	Technology: lack of advanced technology, equipment and infrastructure
Infrastructure	Awareness: lack of public awareness and expertise
Enabling institutions and policies	

As seen in Table 5, the barriers to sustainable waste management implementation were grouped into five categories, namely: cost, legislation, logistics, technology, and lack of public awareness. One of the managers who was interviewed claimed: “*We do not have knowledge, facilities, resources or funds to implement and maintain a CE for the moment. We just dump the waste in landfill sites we have. We do not even separate recyclable waste to some extent”* (Swakopmund P3). In order to completely implement a CE system will include ensuring that the barriers pointed out in Table 5 are minimised while the enablers are improved.

### A proposed conceptual framework for sustainable waste management

**Fig. 6:**
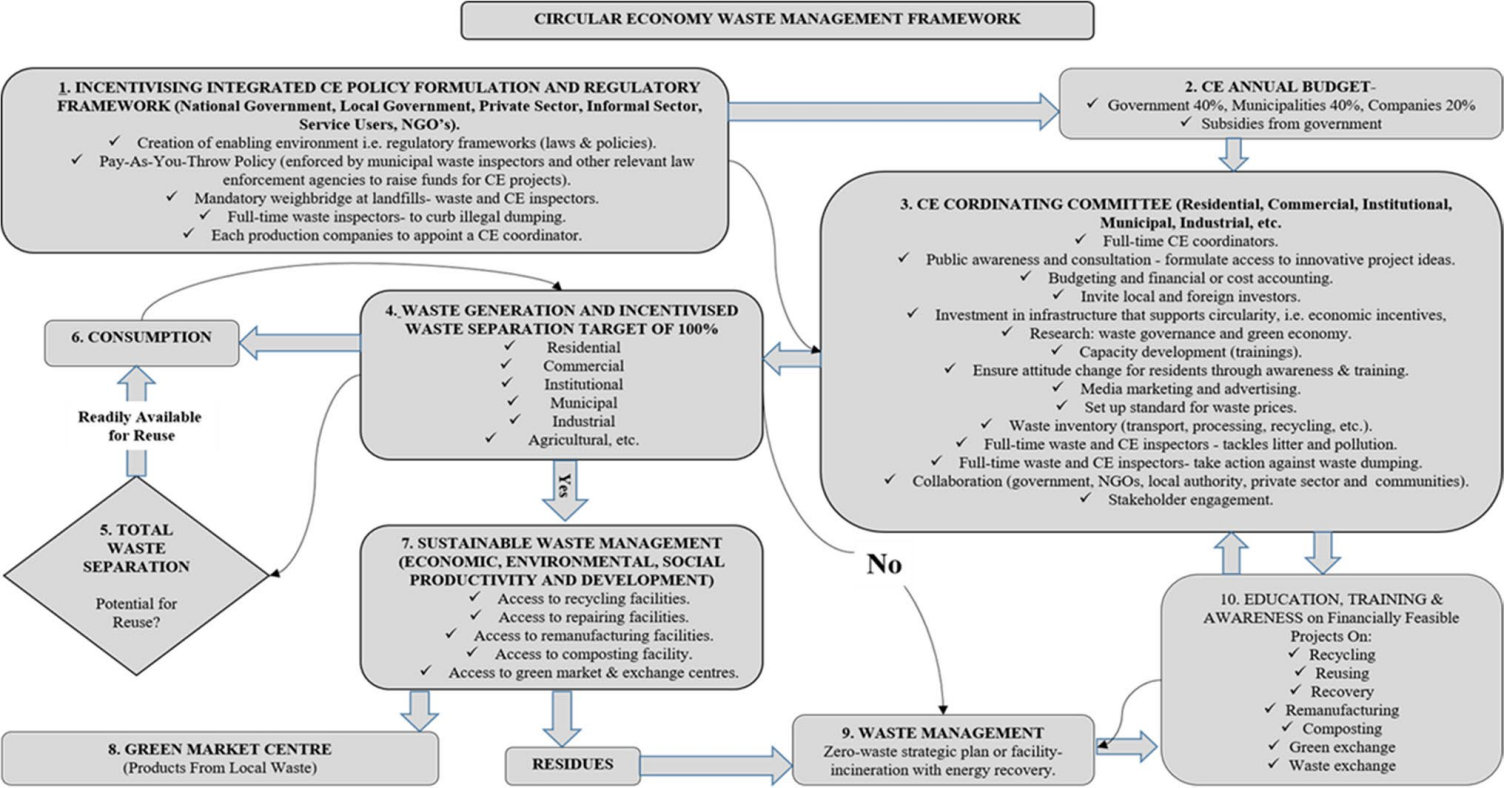
Proposed sustainable waste management conceptual framework—the zero-waste conceptual framework

Figure 6 depicts the developed conceptual framework for sustainable waste management to ensure economic and environmental sustainability and CE principles in small municipalities and to drive action for a zero-waste city. The framework will help to drive action for zero-waste cities and towns for the reason that waste generation will be fully coordinated through a 100% waste separation target. The conceptual framework states that sustainable waste management in a CE occurs when waste is, among others, recovered, reused, repaired, and returned to the consumption mainstream to reduce environmental pollution and degradation. Among the unique aspects in Fig. 6 (the CE zero-waste management conceptual framework) are education, training, and awareness. Hence, it is suggested that launching educational training and awareness campaigns for such initiatives is important because residents are not socially accustomed to waste separation at source. Awareness campaigns can help to influence the social behaviour of inhabitants, who play an important part in defining cities’ and towns’ waste management systems. It is frequently stated that when residents are aware of the advantages and implications of recycling and reuse, among other sustainable measures, they are more inclined to comply with ecologically responsible waste disposal practices (Elsaid and Aghezzaf 2015).

Circularity is achieved through the creation of an enabling environment by establishing regulatory frameworks such as sensitising policies that promote circularity and investment in CE infrastructure, awareness creation through education of all stakeholders, attitude change, and collaboration between all stakeholders such as government, non-governmental organisations, local businesses, and communities. Cost (1) is one of the key barriers to CE implementation; therefore, an annual budget (2) jointly funded by the government (40%), municipality (40%), and stakeholders (20%) should be tabled to run the affairs of the framework successfully. Through the budget, the CE-coordinating committees (3) are appointed to coordinate CE activities and ensure these activities take place at waste generation sources (4).

Waste separation (5) is a crucial step in this ecosystem. Waste which is readily available for reuse is taken back into consumption (6). Waste which has the potential for reuse is recycled, repaired, and remanufactured to be suitable products before being returned to the production cycle (7), and locally manufactured products from waste are sold to local population at green market centres (8) at affordable prices. Only small amounts of residuals that may not be recycled are finally incinerated to produce electricity (9) and to electrify some of the poorest settlements, such as DRC-Swakopmund. Development of the economy is based on this concept, where education, training, and awareness (10) are emphasised among the local community. The primary idea is the effective use of material resources, waste collection, recycling, and reuse in the manufacturing process (Drljača 2015; Upadhayay and Alqassimi 2018). This conceptual framework (Fig. 6) ensures that the waste management goal of zero-waste generation is met. The implementation and application of the proposed conceptual framework (Fig. 6) is not limited to Langebaan and Swakopmund municipalities. Other small municipalities globally that want to fully implement CE concept into their waste management systems may apply this or a similar framework.

### Principles for strategic circular economy implementation

This study suggests that small municipalities must implement the CE model in a strategic manner in order to achieve sustainable development. Sustainable development is development that meets the needs of the present without compromising the ability of future generations to meet their own needs (United Nations World Commission on Environment and Development [UNWCED] 1987).

The following principles are suggested for effective strategic CE implementation:

1. View waste as a resource, and hence efficiently recycle and upcycle these materials to keep their peak value for as long as possible using the proposed CE budget, funded by the government (40%), municipality (40%), and stakeholders (20%). The CE-coordinating committees (comprising representatives of central government, municipality, and stakeholders) should be responsible for drawing up the budget, determining the costs of the CE implementation, and presenting the budget for approval to the government, municipal council, and stakeholders.
2. Make a deliberate strategic choice towards a sustainable development CE, and support CE implementation in cities and towns through financial investment and capacity-building towards CE.
3. Implementation of CE practices is a shared responsibility and must be planned for. There is thus a need for collaboration with government, municipality, and industry.
4. Invest in technology and digitalisation to have innovative business models as an enabling tool to achieve strategic sustainable development within the framework of CE.
5. Strategic sustainable development practices must be aligned to the principles of dependency on renewable energy within the context of a green economy.
6. Through flexible design, diversity, and risk mitigation, the CE system must be built to be resilient and adaptive.

In line with the proposed conceptual framework, municipalities or any other business that are to achieve circularity in sustainable waste management must take the following course of action for strategic CE implementation (Table 6).

**Table 6:** Action for strategic CE implementation

Action	Explanation
Regulatory framework	Policy plays an important role in enabling, or constraining, the transition to a CE. Governments, municipalities, and businesses must develop and implement policy initiatives on CE, aligned with sustainability principles. Implementation of these policies require a change in culture and municipalities must play a significant role in influencing a cultural shift
A systems approach	Transitioning to a CE requires municipalities to go beyond just closing materials, components, and product loops, and promoting sustainability in the way resources are exploited, used, and managed throughout the system. By adopting a systems approach, municipalities must focus on the flow of resources, provision of the required service, governance, infrastructure provision, and innovation
Urban planning	Accelerating the transition to a CE and scaling it up requires removing several barriers. Additionally, circularity does not happen by accident—it has to be planned for. Hence, municipalities are in a strategic position to design, plan, manage, and monitor the city to promote CE principles. Urban planning must also be re-engineered to support circularity
Focus on financial revenue, or savings	Governments and small municipalities should prioritise fiscal instruments into CE initiatives to ensure resource efficiency and investment in enabling infrastructure. To ensure that municipalities become smart cities/towns, there is a need for more investment in CE practices that contribute to potential financial savings or revenues
Need for a collaborative approach	As governments and municipalities are often well-positioned to support and guide the transition to a CE, governments and small municipalities should collaborate with business and industry in their jurisdictions to support the shift to a CE by, for example, enhancing coordination across levels of stakeholders and facilitating technology transfer and resource-sharing in their operations. Moreover, the transition to a CE requires changes for businesses, consumers, and society at large; moving towards a CE is a shared responsibility across all levels of stakeholders such as government, the private sector, and civil society. Therefore, municipalities should take the lead in initiating and implementing educational, informational, and awareness creation programmes to increase awareness and build capacity
Awareness creation	Municipalities must leverage their influence and make information on CE city plans and initiatives easily accessible
Showcasing best practices	Municipalities must develop and implement projects that can inspire and showcase the potential of a CE
Capacity-building	Municipalities must implement capacity-building programmes and develop CE implementation guidelines, as well as support innovative projects geared towards CE
A holistic/systems approach to waste management	Municipalities must not only look at solutions that aim at diverting waste that goes to landfill, but also solve the root causes of those wastes by focusing on upstream solutions. Municipalities, could, for example, work with product manufacturers to adapt their products so that they can be recycled, mended, or reused more efficiently in a local or regional context
Action plans	Municipalities must develop and implement action plans for transitioning to a CE. The action plan lays out a timeframe for production, consumption, waste management, the secondary raw materials market, and sectoral activities on plastics, food waste, essential raw resources, building and demolition, biomass and bio-based materials, innovation and investments, and monitoring
Monitoring and evaluation	There is a need for monitoring approaches to track the implementation of CE. Municipalities should develop and implement monitoring and evaluation frameworks to monitor progress towards transitioning to a CE. Digital technologies open up new possibilities for effective monitoring of the CE. Investing in digital technologies can support the upscaling of the CE and improve its monitoring

## Limitations of the study

The COVID-19 pandemic posed a number of challenges during this study, particularly during data collection. Because of COVID-19 and social-distancing protocols that prohibited close human contact, there was no physical interaction between researchers and participants. All interviews were conducted virtually. Also, due to movement and travel restrictions during the pandemic, direct observation data were only collected in Swakopmund and not in Langebaan. In addition, the study focuses on only two small municipalities in South Africa and Namibia, but there are obviously many other small municipalities in both South Africa and Namibia that were not included in this study. However, the current study’s findings can be extrapolated beyond the two study areas. The proposed framework for small municipalities transitioning to a CE could be applied to nearly any other small municipality seeking circularity in waste management.

## Recommendations

Policy has a significant impact on whether or not the transition to a CE is possible. Therefore, it is recommended that policymakers in small municipalities should revise the existing regulatory instruments to promote transitioning to circular waste management, in line with the proposed waste management framework to promote the CE model. The municipalities of Langebaan and Swakopmund should develop and implement CE policy initiatives that are consistent with sustainability principles. The implementation of these policies necessitates a shift in waste management culture, and municipalities need to play a role in influencing this shift. By doing so, waste materials will be diverted from landfills to waste recovery, recycling, remanufacturing, composting facilities, and exchange centres once the CE policy is implemented.

Governments, Langebaan and Swakopmund municipalities, and other small municipalities need to work in collaboration with businesses and industry in their jurisdictions to support the transition to a CE by, for example, enhancing coordination across all levels of stakeholders, facilitating technology transfer, and resource-sharing in their operations. They need to prioritise fiscal instruments in CE initiatives to ensure resource efficiency and investment in enabling infrastructure.

Langebaan and Swakopmund municipalities need to take the lead in initiating and implementing educational, information, and awareness-creation programmes to increase awareness and build capacity as well as in developing and implementing monitoring and evaluation frameworks to monitor progress towards transitioning to a CE in waste management. It is also recommended that future studies could focus on establishing the costs and effective timeframes of implementing CE waste management frameworks in small municipalities, and on determining the effectiveness of the conceptual framework proposed in this study. The results of the study may serve as the basis for further research to be conducted in Langebaan and Swakopmund, with a specific focus on the costs involved in the implementation of the proposed waste management framework.

## Conclusion

The CE model is gradually gaining momentum as a tool to address sustainable development challenges. The results of this study show that, despite the fact that the CE model is gaining popularity in many cities and towns globally, the waste management systems in Langebaan and Swakopmund municipalities still primarily follow the LE model, with landfilling as the predominant waste disposal method. Therefore, the purpose of this study was to develop a baseline understanding of the current waste management systems in Langebaan and Swakopmund municipalities, and to propose a framework for transforming waste management towards circularity. In this study, a guiding strategic conceptual framework for sustainable waste management is proposed, which would allow the CE-coordinating committee to direct waste management in order to maintain circularity.

## Data Availability

The datasets used and/or analysed during the current study are available from the corresponding author on reasonable request.
